# Clinical recommendations on lung cancer management during the COVID‐19 pandemic

**DOI:** 10.1111/1759-7714.13498

**Published:** 2020-05-29

**Authors:** Yan Xu, Hongsheng Liu, Ke Hu, Mengzhao Wang

**Affiliations:** ^1^ Department of Respiratory and Critical Care Medicine, Peking Union Medical College Hospital Chinese Academy of Medical Sciences and Peking Union Medical College Beijing China; ^2^ Department of Thoracic Surgery, Peking Union Medical College Hospital Chinese Academy of Medical Sciences and Peking Union Medical College Beijing China; ^3^ Department of Radiation Oncology, Peking Union Medical College Hospital Chinese Academy of Medical Sciences and Peking Union Medical College Beijing China

**Keywords:** COVID‐19, lung neoplasms, patient management

## Abstract

Coronavirus disease 2019 (COVID‐19) is spreading worldwide, and has been declared as an international public health concern. Patients with lung cancer are highly susceptible to infection compared to healthy individuals because of systemic immunosuppression induced by malignancy and anticancer therapy. Furthermore, patients with cancer demonstrate poorer outcomes following infection. Hence, patients with lung cancer should be considered a priority group for COVID‐19 prevention. Furthermore, the routine treatment of patients with cancer has been affected during the COVID‐19 pandemic, and patients may not have been able to undergo timely and effective antitumor treatment, thereby indicating a poor prognosis. Here, we provide some suggestions for early identification of COVID‐19 and differential diagnosis in patients with lung cancer who have fever and respiratory symptoms. Our medical team also provide clinical recommendations on lung cancer management during the COVID‐19 pandemic, for carrying out meticulous and individualized clinical management of lung cancer patients and maximum protection to effectively prevent COVID‐19.

**Key points:**

**Significant findings of the study**

This article provides suggestions for early identification of COVID‐19 and differential diagnosis in patients with lung cancer with fever and respiratory symptoms.

**What this study adds**

This article makes clinical recommendations on lung cancer management during the COVID‐19 pandemic.

## Introduction

Coronavirus disease 2019 (COVID‐19) is spreading worldwide, and has been declared as an international public health concern.^1^ It is caused by a novel enveloped RNA betacoronavirus severe acute respiratory syndrome coronavirus 2 (SARS‐CoV‐2).^2^, ^3^, ^4^ On 12 February 2020, the World Health Organization (WHO) officially named the disease caused by this virus as COVID‐19.^1^ To date, the COVID‐19 pandemic is severe and has affected many countries around the world, and more than one million people have been diagnosed with COVID‐19. Various regions have adopted strict control measures. Countries, societies, medical institutions, families and individuals all have a collective role in the fight against COVID‐19.

Lung cancer has the highest mortality and morbidity rates in the world, and there are a considerable number of lung cancer patients.^5^ Due to systemic immunosuppression induced by malignancy and anticancer therapy, patients with lung cancer are highly susceptible to infection compared to healthy individuals. Furthermore, patients with cancer demonstrate poorer outcomes following infection. Hence, patients with lung cancer should be considered a priority group for COVID‐19 treatment and prevention. Furthermore, the routine treatment of patients with cancer has been greatly affected during the COVID‐19 pandemic, and patients may not have been able to undergo timely and effective antitumor treatment, thereby indicating a poor prognosis.^6^ It is, therefore, necessary to carry out meticulous and individualized clinical management for lung cancer patients.^7^, ^8^, ^9^

## COVID‐19

COVID‐19 is a respiratory infectious disease caused by SARS‐CoV‐2.^2^, ^3^, ^4^ Coronaviruses are enveloped, single‐strand, positive‐sense RNA viruses, named as coronaviruses due to the radiating petal‐like or bat‐like protrusions that appear like a crown. Previously, six pathogenic coronavirus subtypes have been detected in humans, of which four subtypes have low pathogenicity and generally cause mild respiratory symptoms, similar to a common cold. However, two subtypes (SARS‐CoV and MERS‐CoV) can cause severe infections, severe acute respiratory syndrome (SARS) and the Middle East respiratory syndrome (MERS), respectively.^10^, ^11^ SARS‐CoV‐2 is a novel virus that has not been previously observed in humans and is currently the seventh coronavirus to cause human infections. The nucleotide homology of the genomes of SARS‐CoV‐2 and SARS‐CoV causing SARS is 79.5%.^4^ Currently, there is a lack of understanding of the origin, characteristics, hosts, and pathogenicity of SARS‐CoV‐2, with knowledge gaps involved in the pathophysiology and treatment of COVID‐19.

Relevant epidemiological history can often be traced in COVID‐19 patients: (i) History of travel or residence in places with local continuous transmission within 14 days before disease onset; (ii) contact with patients presenting fever or respiratory symptoms in communities with reported cases within 14 days before disease onset; (iii) history of contact with COVID‐19 patients within 14 days before disease onset; and (iv) possible disease clusters. However, with the spread of the disease, epidemiological histories of several newly diagnosed patients have become unclear.

Based on the current epidemiological studies, the incubation period of COVID‐19 is usually 1–14 days.^12^ The clinical symptoms primarily include fever, fatigue, and a dry cough, with some patients presenting with nasal congestion, runny nose, and diarrhea.^12^, ^13^, ^14^ In severe cases, patients develop dyspnea, and critical patients rapidly progress to acute respiratory distress syndrome, septic shock, refractory metabolic acidosis, and coagulation disorder.^14^ However, most patients have a mild/moderate disease, with nonpneumonia and mild pneumonia symptoms (80.9%), and around 1.2% of patients are asymptomatic carriers.^15^, ^16^ Most COVID‐19 patients have a good prognosis, and only a few patients develop a critical disease.^16^ Moreover, COVID‐19 deaths mostly occur in elderly people and patients with underlying diseases.^14^, ^16^

## Epidemiological characteristics of cancer patients with comorbid COVID‐19

The Chinese Center for Disease Control and Prevention described and analyzed the epidemiological characteristics of 72 314 patients (including confirmed cases, suspected cases, and clinical diagnostic cases) up to 11 February 2020, in mainland China and observed that among the COVID‐19 patients, 107 (0.5%) were cancer patients, of which six died and thus the crude mortality rate was determined as 5.6%, which is higher than the overall crude mortality rate (2.3%).^16^ A study published by Chinese researchers^17^ analyzed 1590 COVID‐19 patients detailing their medical history records up to 31 January 2020, observing that 18 (1%) presented with a history of cancer, and five patients were diagnosed with lung cancer (5/18, 28%). Moreover, cancer patients demonstrated a higher risk of severe events than noncancer patients (39% vs. 8%, *P* = 0.0003), with symptoms appearing to worsen more rapidly. Dai *et al*.^18^ performed a multicenter study including 105 cancer patients and 536 age‐matched noncancer patients confirmed with COVID‐19. They found COVID‐19 patients with cancer had higher risks in all severe outcomes, and hematological cancer (death rate 33.33%, 3/9), lung cancer (death rate 18.18%, 4/22), or metastatic cancer (stage IV) had the highest frequency of severe events.

Based on these two cohorts, we observed that cancer patients are a susceptible population during the COVID‐19 pandemic with a poor prognosis. We analyzed the possible reasons which included the following: (i) Most cancer patients and their family members make repeated outpatient visits for consultations, with some patients requiring antitumor treatment in hospital.^19^ Therefore, their risk of exposure to COVID‐19 is significantly higher than healthy individuals during the COVID‐19 pandemic; (ii) tumor patients are relatively older and have a lesser understanding of the pandemic compared to young individuals, and have weaker infection prevention and control capabilities; (iii) immunosuppression caused by surgery, radiotherapy, chemotherapy, and immunotherapy is a common occurrence, resulting in the high risk of infection^20^; and (iv) tumor patients relatively have poor physical status, and present with multiple comorbid diseases, especially patients with lung cancer demonstrate comorbid lung disease and poor lung function. When these patients develop comorbid COVID‐19, they usually present with severe symptoms, and their condition may rapidly worsen. Furthermore, the routine treatment of patients with cancer is affected during the COVID‐19 pandemic, and patients may not be unable to undergo timely and effective antineoplastic treatment, thereby indicating a poor prognosis.^21^, ^22^

## Early identification of COVID‐19 and differential diagnosis in patients with lung cancer

In patients with lung cancer, symptoms caused by the tumor itself include coughing, productive cough, dyspnea, and sometimes, fever, etc. Additionally, patients may develop pulmonary treatment‐related adverse events caused by chemotherapy, radiotherapy, immunotherapy, and so on. Furthermore, the chest computed tomography (CT) scans of lung cancer present with a variety of different manifestations. Therefore, the early identification and differential diagnosis of COVID‐19 for lung cancer patients is extremely difficult, necessitating comprehensive examination for differential diagnosis and etiological identification.

### Early identification of COVID‐19

When fever and respiratory symptoms develop or symptoms worsen in patients with lung cancer, they should seek medical attention in the Fever Clinic and undergo sufficient evaluation regarding the risk of COVID‐19.

According to the current WHO recommendations^1^ and the Diagnosis and Treatment Protocol for COVID‐19 (Trial version seven) by the Chinese National Health Commission,^23^, ^24^ a diagnosis of suspected COVID‐19 requires a combination of epidemiological history and clinical manifestation: (i) Fever and/or respiratory symptoms; (ii) imaging presentation of COVID‐19; and (iii) normal or reduced white blood cell count or lymphocytopenia during the early disease stage disease for integrated analysis. Patients who fulfill any of the epidemiological history criteria and any two clinical presentation criteria, or present no clear epidemiological history but fulfill three clinical presentation criteria, are diagnosed as suspected COVID‐19. Suspected COVID‐19 who fulfill any of the following laboratory testing for SARS‐CoV‐2: (i) Positive SARS‐CoV‐2 RT‐PCR testing result; (ii) viral genetic sequence is highly homologous with the known SARS‐CoV‐2; and (iii) positive SARS‐CoV‐2 specific IgM antibody and IgG antibody are considered as confirmed cases.

**Figure 1:**
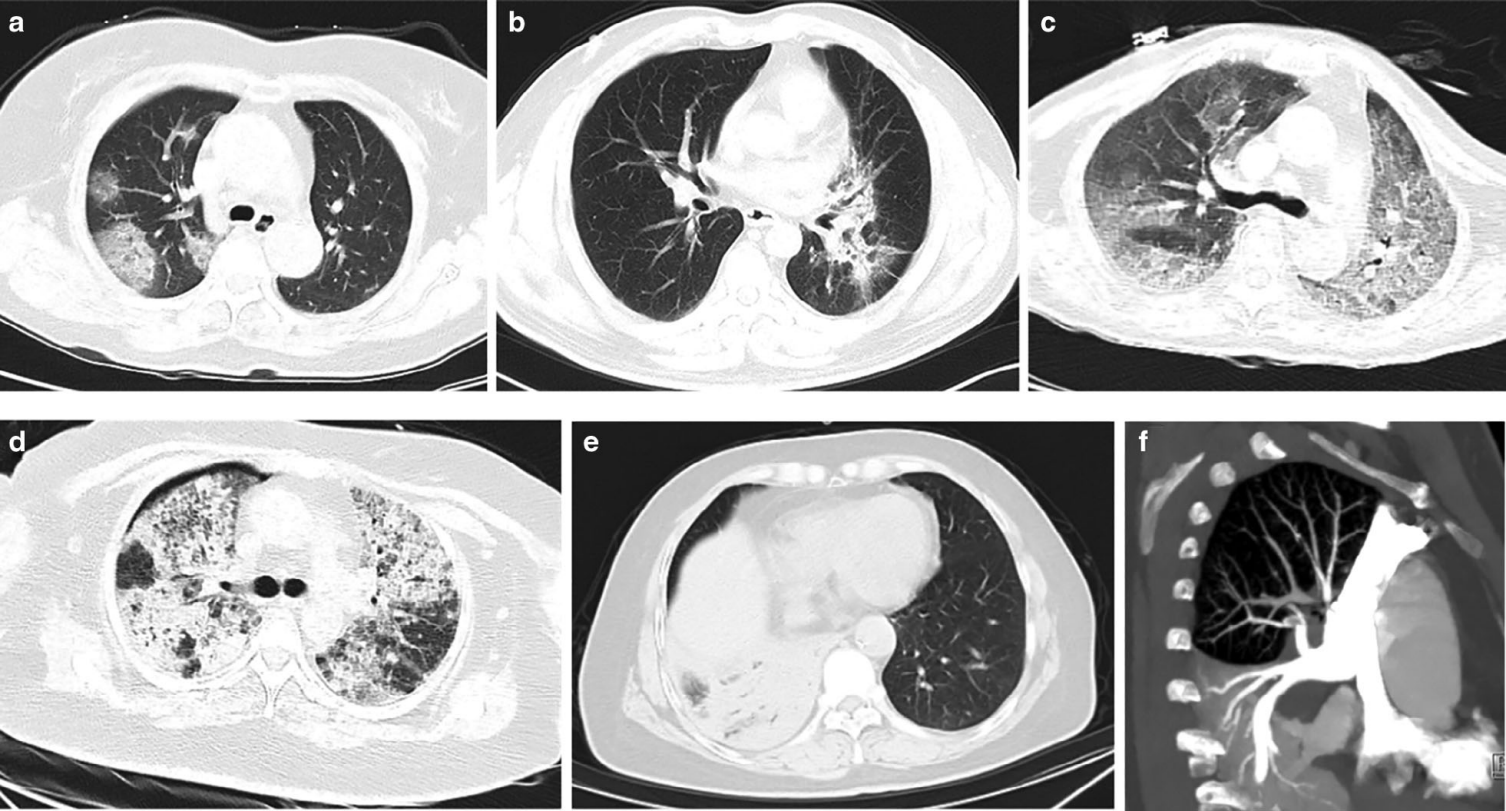
Chest computed tomography (CT )images of COVID‐19 and lung cancer patients. (**a**) Transverse chest CT image from COVID‐19 patient showing ground‐glass opacity (GGO). (**b**) Transverse chest CT image from lung cancer patient with radiation pneumonitis showing GGO consistent with the radiation field. (**c**) Transverse chest CT image from lung cancer patient with ICI‐P showing diffuse GGO. (**d**) Transverse chest CT image from pneumonic‐type lung carcinoma with tumor progression showing diffuse GGO with multiple bubble‐like low attenuation. (**e**) Transverse chest CT image from lung cancer patient with dyspnea showing right lower pulmonary obstructive pneumonia and filling defect of the right superior pulmonary artery on CTPA (**f**) indicating pulmonary embolism.

The patient's history of residence/travel to epidemic regions, related contact history, and infection clusters should be documented in detail. The patient should be questioned in detail regarding recent changes in symptoms. Routine blood, C‐reactive protein (CRP), hepatic and renal function, d‐dimer, creatine kinase, lactate dehydrogenase, and other tests should be completed, and the SARS‐CoV‐2 molecular detection for respiratory tract samples (nasopharyngeal swabs, sputum samples, or lower respiratory tract specimens) and/or SARS‐CoV‐2 serology testing should be performed. CT scans should be obtained to assess pulmonary lesions as soon as possible. If the patient is diagnosed as a suspected or confirmed COVID‐19 patient according to epidemiological history, clinical symptoms, chest CT imaging (Fig 1a), and laboratory testing, the patient must be transferred to a designated hospital for quarantine. If COVID‐19 is excluded, the patient can subsequently receive corresponding treatment or return to the corresponding speciality department for further treatment. However, the possibility of false‐negative results should be considered when nasopharyngeal swabs samples are used for the SARS‐CoV‐2 molecular assays. Therefore, if the first SARS‐CoV‐2 molecular test result is negative but COVID‐19 cannot be clinically ruled out, the patient should undergo another SARS‐CoV‐2 molecular test after a minimum of 24 hours, and sputum, lower respiratory tract secretions, blood, feces, and other samples should be obtained wherever possible for SARS‐CoV‐2 molecular testing. Additionally, SARS‐CoV‐2 specific IgM antibodies and IgG antibodies testing can be used in the differential diagnosis.

### Differential diagnosis

Based on the diagnostic criteria for COVID‐19, patients with an epidemiological history who fulfill any two of the clinical presentation criteria, or no clear epidemiological history but fulfill three of the clinical presentation criteria, are considered as suspected patients.^23^ Therefore, it is extremely important to perform a differential diagnosis in patients with lung cancer to comprehensively analyze the causes for newly developed or worse fever and respiratory symptoms, based on epidemiological history, antitumor treatment, clinical manifestation, chest CT characteristics, laboratory testing for SARS‐CoV‐2, etc.^25^

1. **Pulmonary infections**: these include non‐SARS‐CoV‐2 viral pneumonia (including those caused by influenza virus, respiratory syncytial virus, adenovirus, cytomegalovirus, etc), bacterial pneumonia, mycoplasma pneumonia, legionellosis, *Pneumocystis jirovecii* pneumonia, aspergillus pneumoniae infection, tuberculosis, and so on. Respiratory pathogen detection and pathogen‐specific serology testing should be performed to identify etiology, in order to guiding targeted anti‐infective treatment.
2. **Radiation pneumonitis**: in lung cancer patients undergoing radiotherapy, radiation pneumonitis (Fig 1b) may occur 1–3 months later, and in some patients, during radiotherapy. Patients may present with symptoms of fever and dry cough, and routine blood tests may demonstrate a normal white blood cell count, and chest CT may indicate ground‐glass opacities (GGOs) and patchy shadows.^26^ Moreover, owing to improvements in radiotherapy technology, the characteristics of radiation pneumonitis in imaging have become nonspecific, and hard to differentiate from COVID‐19. A multidisciplinary team comprised of radiation oncologists, oncologists, radiologist, respiratory specialist, and infectious disease specialist is required to comprehensively analyze the radiotherapy regimen and chest CT imaging characteristics, in combination with the specific clinical presentations and laboratory testing results of the patient, for diagnosis differentiation to guide subsequent treatment.
3. **Immune checkpoint inhibitor‐related pneumonitis (ICI‐P)**: patients who receive immunotherapy are at risk of ICI‐P (Fig 1c). The clinical presentation of patients with ICI‐P includes fever, worsening of cough and productive cough, worsening of dyspnea, GGOs and patchy shadows on the chest CT scan, and several patients may present with GGOs in the lungs alone.^27^ Hence this necessitates a multidisciplinary treatment approach formed by the consensus of specialists from various concerned departments.
4. **Tumor progression**: lung cancer progression may result in pneumonic‐type lung carcinoma (Fig 1d), obstructive pneumonitis, lymphangitis carcinomatosa, increased pleural effusion, and pericardial effusion, which may lead to fever and/or worsening of respiratory symptoms. Before and after treatment, chest CT and tumor markers can be used for the differential diagnosis.
5. **Others**: pulmonary embolism (Fig 1e,f), cardiac insufficiency, and immune‐mediated myocarditis can also result in worsening of respiratory symptoms and require related assessment to guide differential diagnosis.

## Clinical management of patients with lung cancer during the COVID‐19 pandemic

Our medical team recommend that patients with lung cancer be managed by considering the following criteria.

### Management of patients with pulmonary nodules

In patients who periodically undergo pulmonary nodule follow‐up consultations, chest CT re‐examination can be delayed during the pandemic. Patients who seek medical attention for fever during the pandemic, as well as CT screening revealed newly pulmonary nodules, must undergo fever diagnosis and treatment according to the procedure of the Fever Clinic. When the fever has improved after treatment, patients should be recommended to quarantine themselves at home for 14 days and avoid immediate outpatient assessment for the pulmonary nodules.

### Scheduled elective surgery for patients with pulmonary nodules or early‐stage lung cancer

1. In patients with suspected malignant pulmonary nodules or early‐stage lung cancer and in those who have completed relevant examinations and are scheduled to undergo elective surgery, the date of the surgery can be delayed due to the COVID‐19 pandemic, particularly in patients with GGOs in the lung, as the short‐term effects on the GGO lesions enlarging are insignificant. Based on individual pulmonary lesions, the specific situation should be analyzed by the physician in charge.^28^ Physicians can maintain communication and follow‐up on patients using the telephone, WeChat, Facebook, or other internet tools during the pandemic, and comprehensively explain the reason for delay in surgery and the risk of COVID‐19.
2. In patients requiring admission for surgery in the foreseeable future or those who need to undergo confirmed procedures, patients are advised to undergo surgery at their local hospital to avoid the possibility of COVID‐19 infection during the journey.
3. If patients who are scheduled for surgery develop fever, cough, productive cough, chest tightness, and shortness of breath, they should seek medical attention at the Fever Clinic. If the patient is asymptomatic but has an epidemiological history, he/she would be required to undergo home quarantine for 14 days, and admission for surgery cannot be performed before the quarantine period is over.

### Postoperative lung cancer patients

In those patients requiring adjuvant chemotherapy after lung cancer surgery, chemotherapy can be performed at a local hospital to avoid traveling if their condition permits. If the tumor stage is graded as IIIa or IIIb based on postoperative pathological staging, with an epidermal growth factor receptor (*EGFR*) gene mutation or ALK rearrangement, oral targeted drug therapy can be considered as a postoperative adjuvant treatment^29^ to avoid chemotherapy‐associated infection and repeated hospital consultations. In patients undergoing routine follow‐up after lung cancer surgery, follow‐up visits can be delayed if the patient's condition is stable. The follow‐up duration is extended based on the epidemic situation.

### Lung cancer patients receiving radiotherapy

1. In the case of patients who have completed radiotherapy localization but have not started radiotherapy, it is recommended that the patients make the following preparations: the patient should stay in the city where the hospital for radiotherapy is located and should avoid traveling as much as possible. If an appointment has been made for treatment at an out‐of‐area hospital, the patient should arrive at that destination 14 days earlier and quarantine himself/herself at home.
2. In the case of patients receiving routine radiation therapy, the following preparations should be made: patients should avoid contact with strangers as much as possible, monitor and record their temperature daily and report temperature to the physician, and pay attention to symptoms such as fever, cough, fatigue, runny nose, and diarrhea. The physician and patient should confirm the time for radiotherapy and arrange an appointment, which can be done through a phone call, or the next radiotherapy date can be confirmed after a radiotherapy session. The patient must undergo consultation and treatment at the radiotherapy department under the premise that personal protective measures have been undertaken. The patient should be present at the consultation punctually at the appointed time and avoid densely concentrated waiting areas. Before radiotherapy, the body temperatures of the patient and family members must be measured. Furthermore, patient details regarding any recent history of fever, history of travel/residence to epidemic regions, and relevant contact history, as well as any recent changes in symptoms, should be determined.
3. Adverse reaction monitoring and assessment during radiotherapy: during radiotherapy, periodic blood routine, hepatic, and renal function tests must be performed. The patient should be questioned regarding recent symptoms, which should be combined with laboratory tests for targeted treatment. During radiotherapy, if the patient develops a fever, cough, dyspnea, or other new symptoms, he/she should seek medical attention at the Fever Clinic of a local hospital. If a patient with lung cancer develops worsening pain and headache but does not present with fever, cough, and dyspnea, he/she can obtain an appointment for a consultation with the outpatient specialist or emergency department of the local hospital.

### Lung cancer patients receiving targeted therapy

1. Patients on oral targeted drug therapy presenting a stable condition should continue taking the prescribed therapeutic drug during the pandemic. If the condition of patients with lung cancer on targeted therapy is stable, the follow‐up consultation and imaging assessment can be delayed based on the patient's condition. Patients receiving regular zoledronic acid injections for bone metastases can delay drug treatment appropriately. To avoid unnecessary travel, patients with lung cancer should seek medical attention at local hospitals as feasible. Patients can make appointments for consultation, undergo telephone follow‐up or through internet consultation. If the patient's condition is stable, family members of lung cancer patients can be requested to bring all medical records and relevant credentials to the hospital to prescribe molecular targeted therapeutic drugs. To reduce the prescription frequency, a longer course of targeted therapeutic drugs can be prescribed according to the policies of the city.
2. If the patient develops a fever, cough, dyspnea, or other new symptoms, he/she should seek medical attention at the Fever Clinic. If a patient with lung cancer develops worsening pain and headache but does not have a fever, cough, and dyspnea, he/she can obtain an appointment for a consultation at the specialist outpatient or emergency department of the local hospital.

### Lung cancer patients receiving chemotherapy and/or immunotherapy

1. Patients should regularly undergo chemotherapy and/or immunotherapy at the nearest hospital to avoid long distance traveling as much as possible. Relevant laboratory tests and examinations can be completed at the outpatient clinic to shorten the treatment duration, and outpatient chemotherapy are recommended for treatment. Due to the epidemic, if patients cannot be admitted for treatment on time, the treatment interval can be extended, or the patient can be switched to oral drugs.
2. Adverse reaction monitoring and assessment during chemotherapy and/or immunotherapy: during chemotherapy and/or immunotherapy, periodic routine blood tests, hepatic, and renal function tests, as well as electrocardiography, must be performed to assess safety. The patient can undergo relevant laboratory tests and examinations at the local hospital. The physician must question the patient regarding symptoms and provide targeted diagnosis and treatment. If the patient develops fever, cough, dyspnea, or other new symptoms, he/she should seek medical attention at the Fever Clinic of a local hospital. If a patient with lung cancer develops worsening pain and headache but does not have fever, cough, and dyspnea, he/she can obtain an appointment for a consultation at the specialist outpatient or emergency department of the local hospital.

### Sufficient and accurate guidance on self‐protection during the pandemic to patients and family members in medical institutions

1. Self‐protection for outpatients: for outpatients, a consultation appointment should be made in advance, and the patient should report for the consultation according to the appointed time to avoid long waiting time at the hospital. The patient should use private transport if possible, and avoid public transport. Patients and their family members should wear masks appropriately during the entire process and avoid using masks with respiratory valves. Patients must undergo consultation in designated zones and avoid walking around the hospital, avoid contact with surfaces and materials in the hospital, and wash their hands immediately after touching. Patients should avoid crowded waiting rooms and maintain a certain distance from other patients. During outpatient consultation, patients must avoid passing through the Fever Clinic and emergency department.
2. Self‐protection during hospitalization: family visits should be avoided or reduced. If a companion is needed, he/she should be a fixed companion who must not exit the premises. During the entire process, patients and their family members should wear surgical masks appropriately, covering their mouths and noses with an elbow or a paper towel when coughing or sneezing, and washing their hands under running water after exposure to respiratory tract secretions. They should also maintain good hand hygiene, including the use of hand soap, soap and running water, or alcohol‐containing hand soap to wash hands after coughing or sneezing, removing masks, following contact with public areas in the hospital, after caring for the patient, before meals and after bowel movements, and after touching contaminated substances. After both hands are thoroughly cleansed, clean towels or paper towels should be used to dry both hands. During hospitalization, patients should not be allowed to leave their wards and move around in other wards. They should reduce contact with other patients and their family members, and maintain a distance from them. Patients should not touch the materials and beds of other patients and their family members.

In summary, with the spread of COVID‐19, patients with cancer, particularly lung cancer patients, are key targets for epidemic control as they will develop severe symptoms after infection with COVID‐19. Furthermore, the mortality rate for COVID‐19 is higher among these patients. Patients with lung cancer receiving antitumor treatment require careful differential diagnosis if they develop a fever and respiratory symptoms to evaluate the risk of COVID‐19. During the COVID‐19 pandemic, precise and individual management is crucial in lung cancer patients, and they should receive maximum protection to effectively prevent COVID‐19.

## Disclosure

The authors declare they have no competing interests.
